# A CT-based radiomics model for predicting pain relief after radiotherapy in patients with bone metastases: a dual-center study

**DOI:** 10.3389/fonc.2026.1813913

**Published:** 2026-04-21

**Authors:** Zhiling Wan, Kangning Liu, Heyao Xu, Fei Zhao, Xiaohan Qin, Zexian Wang, Weijia Li, Yuhang Wu, Bowen Hu, Chong Zhou, Xiaojin Wu

**Affiliations:** 1 The Affiliated Xuzhou Municipal Hospital of Xuzhou Medical University, Xuzhou, China; 2 Department of Radiation Oncology, XuZhou Clinical School of Xuzhou Medical University, Xuzhou, China; 3 Department of Radiation Oncology, Xuzhou Central Hospital, Xuzhou, China

**Keywords:** bone metastasis, computed tomography, machine learning, pain relief, radiomics, radiotherapy

## Abstract

**Purpose:**

This study aimed to develop and validate a CT-based radiomics model for predicting pain relief after palliative radiotherapy in patients with bone metastases, and to compare the performance of 11 machine learning algorithms.

**Methods:**

We retrospectively enrolled patients with bone metastases who received palliative radiotherapy at Xuzhou Central Hospital (Center 1) and Xuzhou First People’s Hospital (Center 2) between January 2022 and December 2024. All patients completed a prescribed dose of 40 Gy in 20 fractions or 30 Gy in 10 fractions. Clinical variables—including age, sex, primary tumor type, pattern of bone destruction, and metastatic site—were collected alongside CT images. Pain response was assessed per the International Consensus on Endpoints for Palliative Radiotherapy in Bone Metastases: complete response (CR) and partial response (PR) were grouped as the relief group, while progressive disease (PD) and stable disease (SD) constituted the non-relief group. ROIs were delineated over the tumor areas on bone-window CT images, and radiomic features were extracted, normalized, and screened to construct a radiomics signature. Eleven machine learning classifiers were trained and compared; the optimal model was selected for predictive performance evaluation and clinical applicability analysis.

**Results:**

A total of 134 eligible patients were included (pain relief group: n = 53; non-relief group: n = 81). Center 1 patients were randomly split approximately 8:2 into training (n = 91) and internal validation (n = 26) sets; Center 2 served as the external test set (n = 17). No significant differences existed between the two centers in baseline demographics, tumor-related variables, or treatment parameters, except for bone-protective drug use and bone metastasis site. After feature selection, 7 radiomic features remained for modeling. Among 11 tested machine learning models, the k-nearest neighbors (KNN) model demonstrated the best performance: area under the receiver operating characteristic curve (AUC) was 0.823 (95% confidence interval (CI): 0.743–0.903) in the training set, 0.812 (95% CI: 0.661–0.964) in the internal validation set, and 0.818 (95% CI: 0.556–1.000) in the external test set. Decision curve analysis (DCA) indicated favorable net clinical benefit.

**Conclusion:**

The KNN model based on CT radiomics can effectively predict pain relief outcomes after palliative radiotherapy in patients with bone metastases, showing potential clinical utility, and may help identify patients likely to achieve pain relief from radiotherapy.

## 1 Introduction

Bone metastasis is the most common distant metastatic manifestation in advanced solid tumors (particularly lung, breast, and prostate cancers) and profoundly compromises survival and quality of life. Skeletal-related events (SREs), including pain, pathological fracture, vertebral compression, spinal cord compression, and hypercalcemia, are frequent sequelae (1, 2). Given the complex pathogenesis of bone metastasis and its rapid disease progression, effective pain relief and improvement of quality of life remain critical challenges in palliative oncology.

Radiotherapy, as an important treatment modality for pain relief in bone metastases, is widely used in palliative care and can significantly alleviate pain, promote bone stabilization, and improve functional status (3). Conventional external beam radiation therapy (EBRT) provides effective pain relief for bone metastases, achieving an overall response rate of 72%–75% and a complete response rate of 28%–30% among evaluable patients (4, 5).However, substantial heterogeneity exists in the definitions of pain response adopted across the trials in the aforementioned studies. A meta-analysis by Imano et al., based on the International Consensus Pain Response Endpoints (ICPRE) criteria, reported an overall response rate of 60.4% and a complete response rate of 12.9% among evaluable patients, both of which are lower than the results from previous meta-analyses that utilized non-standardized criteria (6).In contrast, a large-scale real-world prospective cohort study utilizing the same ICPRE criteria reported an overall response rate of 70% and a complete response rate of 24%, which are higher than the findings of the aforementioned meta-analysis, suggesting considerable variation in response rates within real-world settings (7).There is no significant difference in pain response between different fractionation regimens (e.g., a single 8-Gy fraction versus multiple fractions) (4, 5, 7).In addition to conventional radiotherapy, stereotactic body radiotherapy(SBRT) has demonstrated higher local control rates and favorable pain relief outcomes in selected lesions; however, approximately 40% of patients still fail to achieve satisfactory pain relief following radiotherapy (6, 8, 9).

Current clinical prediction models, based on World Health Organization (WHO) performance status, baseline pain scores, or inflammatory markers (e.g., C-reactive protein), exhibit limited discriminative power for individualized therapy planning (10). In contrast, radiomics enables high-throughput extraction of quantitative imaging features (e.g., texture, shape, intensity) from Computed Tomography (CT), Magnetic Resonance Imaging (MRI), or Positron Emission Tomography scans (PET), reflecting intratumoral heterogeneity and microenvironmental states (11–14). Several studies have shown that radiomics can predict radiotherapy response, local recurrence, and toxicity, which profoundly affect patient survival and quality of life (15). For instance, Cheung et al. developed a CT-radiomics model using skewness and root-mean-square features to predict radiological response after SBRT in early-stage non-small-cell lung cancer (NSCLC) and oligometastatic disease (AUC > 0.8) (16); Lou et al. integrated CT-radiomics with clinical variables to personalize SBRT dosing in 944 lung cancer patients (12). Similarly, Liu et al. achieved an AUC of 0.976 for predicting pathological complete response (pCR) in locally advanced rectal cancer using pre- and post-neoadjuvant chemoradiotherapy MRI radiomics (17).

Despite these advances, radiomics applications in bone metastasis pain relief prediction remain scarce, with prior studies hampered by small sample sizes, inadequate validation, or limited algorithmic comparison (18–21).

To address this gap, we conducted a dual-center study with independent external validation, systematically evaluating 11 machine learning algorithms to establish a robust, generalizable radiomics model for predicting radiotherapy-induced pain relief.

## 2 Materials and methods

### 2.1 Study population

**Figure 1:**
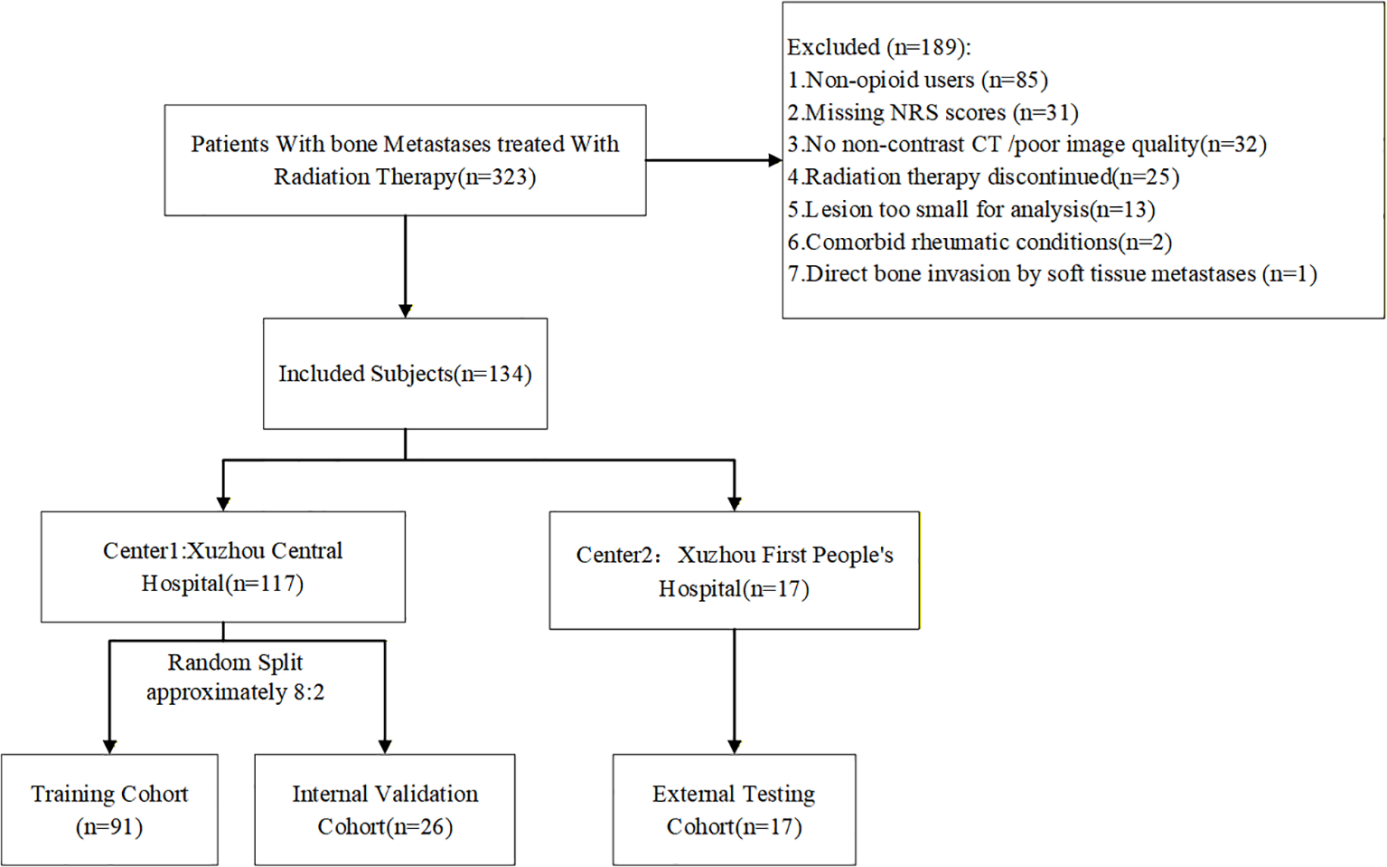
Flowchart for patient recruitment.

The institutional review board approved this study, and patient informed consent was waived due to its retrospective nature. A total of 323 patients with bone metastases who received palliative radiotherapy at Xuzhou Central Hospital and Xuzhou First People’s Hospital between January 2022 and December 2024 were enrolled, and 134 patients were included after screening. Inclusion criteria (1): histologically confirmed malignancy with imaging-verified bone metastasis (2); pain prior to radiotherapy requiring opioid analgesia; (3) availability of Numeric Rating Scale (NRS) score documentation; (4) age > 18 years; (5) complete and detailed medical records. Exclusion criteria: (1) concurrent non-malignant skeletal disorders such as bone tuberculosis, rheumatoid arthritis, or osteoporotic fracture; (2) history of other chronic pain conditions (e.g. lumbar disc herniation, migraine) precluding accurate attribution of the pain etiology; (3) Voluntary withdrawal from radiotherapy, treatment intolerance, or failure to receive the full prescribed dose; (4) absence of CT images or poor image quality severely affecting region of interest (ROI) delineation and feature extraction; (5) lesions resulting from soft tissue metastasis directly invading bone rather than true bone metastasis; (6) lesion volume too small (< 1 cm³) (22). The enrollment and screening flowchart is shown in **Figure 1**.

Clinical variables of patients with bone metastases were obtained by reviewing medical records, including gender, age, clinical diagnosis, radiotherapy site, bone destruction type (mixed predominantly osteolytic/mixed predominantly osteoblastic), primary tumor categories, metastasis time (from primary tumor diagnosis to bone metastasis diagnosis), SREs (pathological fracture, spinal cord compression), bone metastasis site, number of bone metastases, bone-protective drugs, pre-treatment Karnofsky Performance Status (KPS) scores, and pre-treatment NRS scores. Radiotherapy Treatment Planning: At Center 1, treatment plans were delivered on a TrueBeam linear accelerator, while at Center 2, plans were executed on a Vital Beam linear accelerator. Volumetric modulated arc therapy (VMAT) was employed using 6 MV photon beams, with either single-arc or dual-arc delivery modes. The prescribed dose was 40 Gy in 20 fractions (2.0 Gy per fraction) or 30 Gy in 10 fractions (3.0 Gy per fraction). The maximum dose to the spinal cord, as an organ at risk (OAR), was strictly limited to below 45 Gy.

### 2.2 Pain assessment

To quantitatively evaluate the analgesic efficacy of radiotherapy, pain assessment in this study was conducted according to the ICPRE (23), using the NRS combined with Morphine Milligram Equivalents (MME; MME is a standardized metric used to quantify the cumulative potency of various opioid prescriptions.) The MME conversion factors were referenced from the conversion table provided by the Centers for Disease Control and Prevention (CDC) (**Supplementary Table 1**), which includes detailed information on opioid medications (including routes of administration, such as oral or transdermal) (24).Clinical Data Collection and Radiotherapy Planning.

### 2.3 Efficacy evaluation criteria

(1) Pain and performance status assessment: NRS scores and KPS scores were each evaluated before radiotherapy and at 1 month after completion of radiotherapy. The NRS has a total score of 10, with higher scores indicating more severe pain (25); the KPS has a total score of 100, with higher scores indicating better performance status (26).

(2) Response assessment: according to the “International Consensus Pain Relief Endpoints (ICPRE)”,pain response was retrospectively graded based on patient records (23), classified as follows: Complete response (CR): post-treatment NRS score of 0, with no increase in opioid dosage; Partial response (PR): post-treatment NRS score decreased by ≥ 2 points compared with pre-treatment, with no increase in opioid dosage; Progressive disease (PD): post-treatment NRS score increased by ≥ 2 points compared with pre-treatment with no reduction in opioid dosage, or opioid dosage increased by ≥ 25% (regardless of changes in NRS score); Stable disease (SD): no significant changes in either opioid dosage or post-treatment NRS score. In this study, CR and PR were defined as the relief group, while SD and PD were defined as the non-relief group.

### 2.4 CT simulation positioning and image acquisition

Patients were positioned supine using a Klarity R616 integrated immobilization frame with a hip cushion and thermoplastic body mask for thorough immobilization to prevent positional movement. Xuzhou Central Hospital used a large-bore CT simulator (Siemens SOMATOM Definition AS 40, Germany), and Xuzhou First People’s Hospital used a 16-slice large-bore spiral CT simulator (Philips Brilliance Big Bore CT). Scanning parameters: tube voltage 120 kV, tube current 240 mAs, the laser alignment mark was positioned near the center of the bone metastasis, patients breathed freely and slowly during CT scanning, and the slice thickness was 5 mm.

### 2.5 Delineation of bone metastasis target volume

Non-contrast CT images were saved in Digital Imaging and Communications in Medicine (DICOM) format and imported into 3D Slicer software (version 5.1.0, https://www.slicer.org/) for manual slice-by-slice segmentation of lesions on axial non-contrast CT images to encompass the entire lesion. Tumor boundaries were delineated based on the bone window setting, and for tumor boundaries that were difficult to identify on non-contrast CT images, determination was made with reference to additional MRI images and clinical experience. For cases with multiple metastatic lesions, only the largest lesion was selected for analysis. ROI delineation was performed by a single radiation oncologist (Dr. Chong Zhou) with over 10 years of experience in both diagnostic imaging and radiation oncology. All contours were subsequently reviewed, modified, and validated by a senior radiation oncology professor (Prof. Xiaojin Wu) with more than a decade of clinical practice and extensive expertise in diagnostic imaging and target volume delineation. The delineated CT images and ROI images were saved in NIfTI (Neuroimaging Informatics Technology Initiative) format (https://www.nitrc.org/projects/nifti/). To evaluate the reproducibility of radiomics feature extraction, 25 cases were randomly selected from the entire cohort. A radiation oncologist, blinded to the original segmentations, independently re-delineated the regions of interest (ROIs) using the same 3D Slicer software with identical bone window settings. Radiomics features were subsequently extracted using the same PyRadiomics parameters, and the intraclass correlation coefficient (ICC) was calculated for each feature.

### 2.6 Feature extraction

Feature extraction was performed using the PyRadiomics package (version 3.0.1, https://pyradiomics.readthedocs.io/en/latest/). To reduce confounding factors, image normalization and resampling with uniform parameters were conducted prior to feature extraction. The names of all extracted features and computational methods conformed to the specifications outlined in the Image Biomarker Standardization Initiative (IBSI, https://ibsi.radiomics.hevs.ch/).

All computed tomography images and their corresponding region-of-interest masks were resampled to isotropic voxels of 3 × 3 × 3 mm using the nearest-neighbor interpolation algorithm, with a padding distance of 10 applied at the image boundaries, followed by geometric correction of the masks. Image intensities were normalized by shifting Hounsfield unit values by 1000 to eliminate negative values and then scaling them to a range of 0–1000. Subsequently, a fixed gray-level discretization strategy was applied with a bin width of 5 for intensity quantization. Features were extracted from both the original images and eight types of preprocessed images, including Laplacian of Gaussian filtering (σ = 1.0, 2.0, and 3.0), wavelet transformation, three-dimensional local binary pattern, exponential, square, square root, logarithm, and gradient transformations. The extracted features comprised seven major categories: shape, first-order statistics, gray level co-occurrence matrix (GLCM), gray level run length matrix(GLRLM), gray level size zone matrix(GLSZM), gray level dependence matrix(GLDM), and neighboring gray tone difference matrix(NGTDM).

### 2.7 Feature selection

First, Z-score normalization to a standard normal distribution (mean of 0, variance of 1) was performed on all 1,834 radiomic features across all cases. Feature selection consisted of the following four steps:(1) Difference testing: First, the Shapiro–Wilk test was used to assess the normality of each radiomic feature. For features that conformed to a normal distribution, Student’s t-test was used for between-group comparisons; for features that did not conform to a normal distribution, the Mann–Whitney U test was used for between-group comparisons. Features showing significant differences between the pain relief group and the non-relief group were identified using p < 0.05 as the screening criterion. (2) Delineation consistency: Based on the inter-observer reproducibility assessment, only radiomic features with an ICC > 0.75 were selected for subsequent analysis.(3) Correlation analysis: Pearson correlation coefficients were used to evaluate inter-feature correlations. During this process, a greedy recursive elimination method was applied for feature filtering; for any pair of features with a correlation coefficient exceeding 0.9, one was randomly eliminated, with 10 of the most redundant features removed per iteration. (4) Construction of a Least Absolute Shrinkage and Selection Operator (LASSO) regression model: Based on the minimum error probability criterion, the parameter λ with the minimum error was determined through 10-fold cross-validation.

### 2.8 Machine learning model development and validation

Eleven classifiers were trained: Base learners: logistic regression (LR), Naïve Bayes (NB), k-nearest neighbors (KNN), support vector machine (SVM), multilayer perceptron (MLP); Ensemble methods: random forest (RF), extra trees (ET), AdaBoost, gradient boosting (GB), XGBoost, LightGBM.

Models were trained on the Center 1 training set (n = 91), internally validated on Center 1 validation set (n=26), and externally tested on Center 2 (n=17). Performance was evaluated using AUC, accuracy, sensitivity, specificity, F1-score, positive/negative predictive value (PPV/NPV). DCA assessed clinical net benefit.

**Figure 2:**
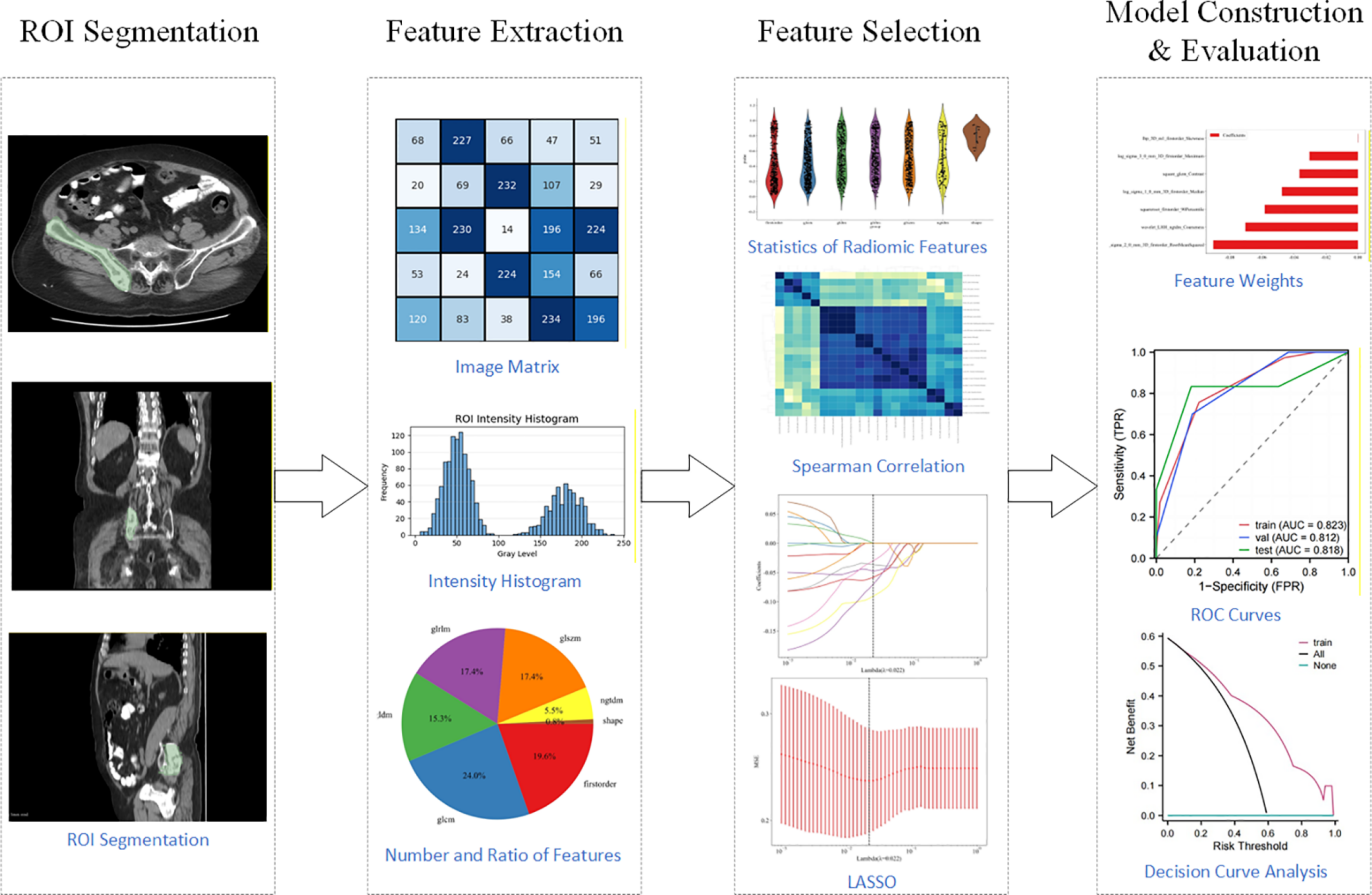
Workflow of radiomics analysis and prediction in this study.

The complete radiomics workflow is illustrated in **Figure 2**.

### 2.9 Statistical methods

Analyses used SPSS v27.0 (IBM), R v4.2.1, and Python v3.7.12. The scikit-learn library (v1.0.2) in Python was used for prediction model construction and validation. Continuous variables were tested for normality (Shapiro–Wilk); normally distributed data were presented as mean ± SD and compared via Student’s t-test; non-normal data as median (IQR, Interquartile Range) [M(IQR)] and compared via Mann–Whitney U test. Categorical variables were expressed as proportions and compared using χ² or Fisher’s exact test. Statistical significance was set at a two-sided p value < 0.05.

## 3 Results

### 3.1 Baseline clinical characteristics of patients

A total of 134 patients with bone metastases who received palliative radiotherapy were ultimately included in this study. According to the aforementioned efficacy evaluation criteria, 53 patients achieved pain relief (CR+PR) and were classified into the relief group, while the remaining 81 patients exhibited stable disease or progression (SD+PD) and were classified into the non-relief group. Patients from Center 1 were randomly divided in an approximately 8:2 ratio into a training set (n=91) and an internal validation set (n=26), while Center 2 served as the external test set (n=17). All baseline clinical characteristics were compared between Center 1 and Center 2 (**Table 1**). Statistical analysis showed that, except for bone-protective drugs and bone metastasis site, there were no statistically significant differences between the two groups in demographic characteristics, tumor-related features, or treatment-related factors (all P > 0.05).

**Table 1:** The baseline characteristic comparison results of the two centers.

Variables	Xuzhou First People's Hospital (n = 17)	Xuzhou Central Hospital (n = 117)	*P*
Age, Mean ± SD	61.65 ± 10.50	62.62 ± 10.66	0.724
Metastasis Time (months), Median (IQR)	2 (0, 15)	6 (0, 24)	0.375
Pre-treatment KPS score, Median (IQR)	70 (50, 100)	80 (70, 95)	0.313
Pre-treatment NRS score, Median (IQR)	3 (2, 4)	2 (2, 4)	0.140
Gender, n(%)			0.374
Male	9 (52.9%)	75 (64.1%)	
Female	8 (47.1%)	42 (35.9%)	
Radiotherapy Site, n (%)			0.716
Spine	12 (70.6%)	82 (70.1%)	
Pelvic	3 (17.6%)	22 (18.8%)	
Extremities	2 (11.8%)	7 (6.0%)	
Others	0 (0.0%)	6 (5.1%)	
Bone Destruction Type, n (%)			0.984
Mixed Lesion with Predominant Osteoblastic	11 (64.7%)	76 (65.0%)	
Mixed Lesion with Predominant Osteolysis	6 (35.3%)	41 (35.0%)	
Skeletal-related events, n (%)			0.690
None	11 (64.7%)	81 (69.2%)	
Pathological Fracture	3 (17.6%)	23 (19.7%)	
Spinal Cord Compression	3 (17.6%)	10 (8.5%)	
Combined	0 (0.0%)	3 (2.6%)	
Bone Metastasis Site, n (%)			0.049
Axial Skeleton	13 (76.5%)	91 (77.8%)	
Peripheral Skeleton	2 (11.8%)	1 (0.9%)	
Mixed	2 (11.8%)	25 (21.4%)	
Number of Bone Metastases, n (%)			0.099
1	4 (23.5%)	55 (47.0%)	
2	6 (35.3%)	36 (30.8%)	
3	6 (35.3%)	16 (13.7%)	
>3	1 (5.9%)	10 (8.6%)	
Bone-protective Drugs, n (%)			<0.001
None	3 (17.6%)	44 (37.6%)	
Denosumab	12 (70.6%)	30 (25.6%)	
Bisphosphonates	2 (11.8%)	43 (36.8%)	
Primary Tumor Categories, n (%)			0.332
Lung cancer	6 (35.3%)	59 (50.4%)	
Prostate cancer	1 (5.9%)	12 (10.3%)	
Breast cancer	1 (5.9%)	8 (6.8%)	
Liver cancer	1 (5.9%)	6 (5.1%)	
Renal cell carcinoma	0 (0.0%)	7 (6.0%)	
Colorectal cancer	2 (11.8%)	10 (8.5%)	
Others	6 (35.3%)	15 (12.8%)	

This study presents the detailed baseline characteristics of the overall population (N = 134) (**Table 2**) to provide a comprehensive overview of the target patient population, rather than separately comparing the statistical differences among individual subcohorts.

**Table 2:** Baseline characteristics of patients.

Characteristics	Relief group (n=53)	Non-relief group (n=81)	P value
Age (years), Mean ± SD	62.60 ± 11.26	62.43 ± 10.23	0.927
Gender,n(%)			0.168
Male	37 (69.8%)	47 (58.0%)	
Female	16 (30.2%)	34 (42.0%)	
Pre-treatment KPS score, Median (IQR)	80 (65, 95)	85 (70, 100)	0.245
Pre-treatment NRS score, Median (IQR)	2 (2, 4)	2 (1, 4)	0.881
Primary Tumor Categories, n (%)			0.247
Lung cancer	24 (45.3%)	41 (50.6%)	
Prostate cancer	8 (15.1%)	5 (6.2%)	
Breast cancer	1 (1.9%)	8 (9.9%)	
Liver cancer	4 (7.5%)	3 (3.7%)	
Renal cell carcinoma	2 (3.8%)	5 (6.2%)	
Colorectal cancer	4 (7.5%)	8 (9.9%)	
Others	10 (18.9%)	11 (13.6%)	
Metastasis Time (months), Median (IQR)	9 (0, 20)	5 (0, 25)	0.911
Bone Metastasis Site, n (%)			0.968
Axial Skeleton	41 (77.4%)	63 (77.8%)	
Peripheral Skeleton	1 (1.9%)	2 (2.5%)	
Mixed	11 (20.8%)	16 (19.8%)	
Number of Bone Metastases, n (%)			0.575
1	15 (28.3%)	27 (33.3%)	
2	11 (20.8%)	11 (13.6%)	
3	3 (5.7%)	8 (9.9%)	
>3	24 (45.3%)	35 (43.2%)	
Bone Destruction Type, n (%)			0.184
Mixed Lesion with Predominant Osteoblastic	38 (71.7%)	49 (60.5%)	
Mixed Lesion with Predominant Osteolysis	15 (28.3%)	32 (39.5%)	
Skeletal-related events, n (%)			0.515
None	36 (67.9%)	56 (69.1%)	
Pathological Fracture	11 (20.8%)	15 (18.5%)	
Spinal Cord Compression	6 (11.3%)	7 (8.6%)	
Combined	0 (0.0%)	3 (3.7%)	
Radiotherapy Site, n (%)			0.424
Spine	39 (73.6%)	55 (67.9%)	
Pelvic	11 (20.8%)	14 (17.3%)	
Extremities	2 (3.8%)	7 (8.6%)	
Others	1 (1.9%)	5 (6.2%)	
Bone-protective Drugs, n (%)			0.134
None	24 (45.3%)	23 (28.4%)	
Denosumab	14 (26.4%)	28 (34.6%)	
Bisphosphonates	15 (28.3%)	30 (37.0%)	

Statistical analysis of **Table 2** revealed no statistically significant differences between the relief group and the non-relief group in age or sex distribution. Furthermore, tumor-related characteristics, including primary tumor type, site of bone metastasis, number of bone metastases, type of bone destruction, time interval between diagnoses, skeletal-related events, radiotherapy site, treatment-related factors including bone-protective agent treatment status, pre-treatment KPS scores, and pre-treatment NRS scores, showed no statistically significant differences in distribution between the two groups (all P > 0.05). These results indicated that conventional clinical characteristics could not effectively predict pain relief outcomes following radiotherapy.

### 3.2 Radiomic feature extraction and selection

**Figure 3:**
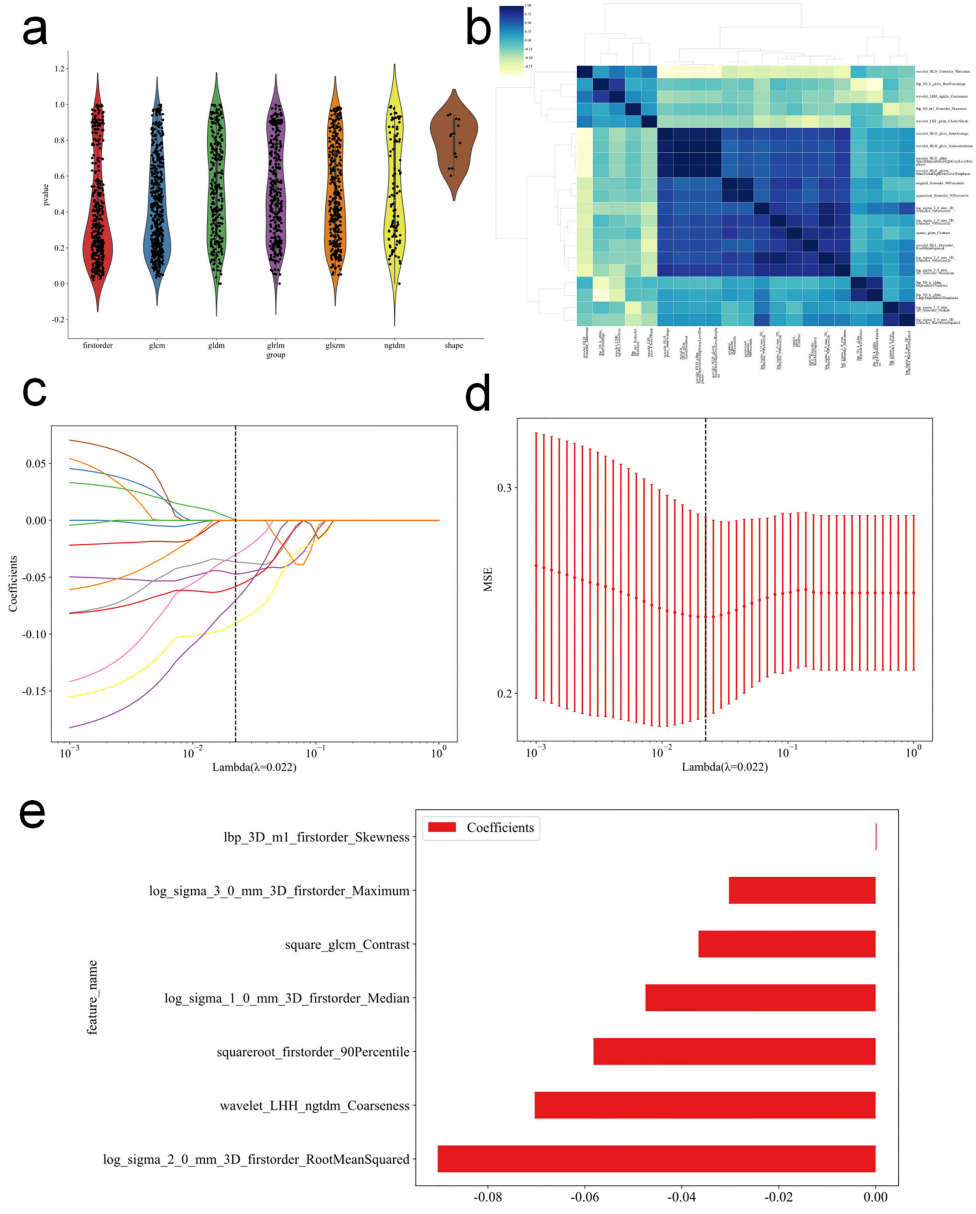
Radiomics feature selection and signature construction workflow. **(A)** Violin plots illustrating the distribution of P values across different feature categories (e.g., Shape, First Order, GLCM). This provides an overview of the statistical significance of features prior to selection. **(B)** Cluster heatmap showing the correlation matrix of radiomics features, indicating high redundancy among extracted features. **(C)** LASSO coefficient profiles of the radiomics features. **(D)** Selection of the optimal tuning parameter (λ) in the LASSO model using 10-fold cross-validation. The vertical dashed line indicates the optimal λ (0.022) with the minimum mean squared error (MSE). **(E)** Histogram of the final selected radiomics features and their corresponding coefficients, which constitute the radiomics signature.

First, a total of 1,834 radiomic features were extracted from the ROI of each patient. After Z-score normalization of all features, four steps were performed for feature selection. In the first step, 21 potential features were preliminarily identified through t-tests or U-tests (**Figure 3A**).In the second step, based on the inter-observer reproducibility assessment, all 21 features demonstrated ICCs > 0.75 and were therefore retained. In the third step, redundant features were eliminated using Pearson correlation coefficients and greedy recursive elimination, leaving 14 features (**Figure 3B**). In the fourth step, the LASSO regression algorithm was applied to select the optimal feature subset (**Figures 3C, D**). Ultimately, 7 radiomic features were identified for constructing the radiomics signature.

The seven radiomic features ultimately selected in this study were all extracted from filtered and transformed images rather than from the original images, and were therefore considered higher-order features. In terms of basic radiomic feature categories, five were derived from first-order statistics (skewness, median, root mean square, maximum, and 90th percentile), whereas two were derived from texture matrices (GLCM contrast and NGTDM coarseness). In terms of filter type, these features were obtained from Laplacian of Gaussian-filtered images (three features; σ = 1.0, 2.0, and 3.0), three-dimensional local binary pattern-transformed images (one feature), square-transformed images (one feature), square root-transformed images (one feature), and wavelet-transformed images (one feature).

Based on the selected features and their corresponding LASSO regression coefficients, the radiomics score (Rad-score) for each patient was calculated using a linear weighted combination (**Figure 3E**), with the formula as follows (Formula 1):


$$\begin{array}{c} Rad-score\;=\;0.407\;+\;0.0002\;\times \;lbp\_3Dm1\_Skewness\\ -\;0.048\;\times \;log\_sigma\_1\_0\_Median\;-\;0.090\\ \;\times \;log\_sigma\_2\_0\_RMS\;-\;0.030\times \\ \;log\_sigma\_3\_0\_Maximum\;-\;0.037\\ \;\times \;square\_glcm\_Contrast\\ -\;0.058\;\times \;squareroot\_90Percentile\;-\;0.070\;\\ \times \;wavelet\_LHH\_ngtdm\_Coarseness \end{array}$$

(Note: Feature names in the formula are abbreviated; the detailed feature list and parameters are provided in **Supplementary Table 2**).

### 3.3 Performance comparison of different machine learning models

The detailed performance metrics of 11 mainstream machine learning classifiers across the training set, internal validation set, and test set are presented in **Table 3**. The results revealed substantial differences in performance among the various classifiers. Ensemble tree models (such as XGBoost, Gradient Boosting, and AdaBoost), despite achieving extremely high predictive accuracy in the training cohort, exhibited marked performance degradation in the validation and test cohorts (AUC declining to the 0.6–0.7 range), suggesting evident overfitting. Although logistic regression ranked second only to the KNN model, its perfect specificity and PPV were more attributable to the incidental correct classification of a small number of negative samples rather than robust discriminative ability. In contrast, the KNN model demonstrated the best overall performance and generalization stability. In the training set, the KNN model achieved an AUC of 0.823 (95% CI: 0.743–0.903). In the validation set and test set, the model maintained robust discriminative capability, with AUCs of 0.812 (95% CI: 0.661–0.964) and 0.818 (95% CI: 0.556–1.000), respectively. Additionally, the KNN model achieved an accuracy of 82.4%, a sensitivity of 83.3%, and a specificity of 81.8% in the test cohort. Based on the above results, clinical factors were not incorporated into the final model construction, and the KNN classifier was ultimately selected as the optimal radiomics classifier for subsequent analysis in this study.

**Table 3:** The performance of different machine-learning models.

Model name	Cohort	AUC	95% CI	Accuracy	Sensitivity	Specificity	PPV	NPV	F1
LR	train	0.757	0.6592-0.8543	0.670	0.919	0.500	0.557	0.900	0.694
	val	0.744	0.5468-0.9407	0.692	0.800	0.625	0.571	0.833	0.667
	test	0.864	0.5934-1.0000	0.941	0.833	1.000	1.000	0.917	0.909
Naive Bayes	train	0.736	0.6351-0.8364	0.626	1.000	0.370	0.521	1.000	0.685
	val	0.712	0.5060-0.9190	0.615	1.000	0.375	0.500	1.000	0.667
	test	0.712	0.4462-0.9781	0.765	0.667	0.818	0.667	0.818	0.667
SVM	train	0.751	0.6499-0.8516	0.703	0.838	0.611	0.596	0.846	0.697
	val	0.744	0.5505-0.9370	0.654	1.000	0.437	0.526	1.000	0.690
	test	0.788	0.5103-1.0000	0.824	0.667	0.909	0.800	0.833	0.727
KNN	train	0.823	0.7430-0.9026	0.769	0.757	0.778	0.700	0.824	0.727
	val	0.812	0.6611-0.9639	0.769	0.700	0.812	0.700	0.812	0.700
	test	0.818	0.5560-1.0000	0.824	0.833	0.818	0.714	0.900	0.769
Random Forest	train	0.881	0.8115-0.9502	0.824	0.784	0.852	0.784	0.852	0.784
	val	0.575	0.3378-0.8122	0.615	0.700	0.562	0.500	0.750	0.583
	test	0.750	0.4744-1.0000	0.765	0.667	0.818	0.667	0.818	0.667
Extra Trees	train	0.952	0.9145-0.9894	0.879	0.919	0.852	0.810	0.939	0.861
	val	0.769	0.5821-0.9554	0.692	1.000	0.500	0.556	1.000	0.714
	test	0.697	0.3666-1.0000	0.824	0.667	0.909	0.800	0.833	0.727
XG Boost	train	0.983	0.9623-1.0000	0.945	0.973	0.926	0.900	0.980	0.935
	val	0.641	0.4045-0.8768	0.654	0.800	0.562	0.533	0.818	0.640
	test	0.583	0.2715-0.8952	0.647	0.667	0.636	0.500	0.778	0.571
LightGBM	train	0.655	0.5626-0.7477	0.615	0.811	0.481	0.517	0.788	0.632
	val	0.488	0.3205-0.6545	0.385	1.000	0.000	0.385	0.000	0.556
	test	0.780	0.5665-0.9941	0.765	0.833	0.727	0.625	0.889	0.714
Gradient Boosting	train	1.000	1.0000-1.0000	1.000	1.000	1.000	1.000	1.000	1.000
	val	0.681	0.4606-0.9019	0.654	0.900	0.500	0.529	0.889	0.667
	test	0.576	0.2307-0.9208	0.706	0.500	0.818	0.600	0.750	0.545
Ada Boost	train	0.899	0.8386-0.9587	0.835	0.676	0.944	0.893	0.810	0.769
	val	0.700	0.4806-0.9194	0.654	0.800	0.562	0.533	0.818	0.640
	test	0.583	0.2885-0.8782	0.706	0.833	0.636	0.556	0.875	0.667
MLP	train	0.763	0.6668-0.8588	0.659	0.946	0.463	0.547	0.926	0.693
	val	0.669	0.4470-0.8905	0.692	0.500	0.812	0.625	0.722	0.556
	test	0.712	0.3849-1.0000	0.824	0.667	0.909	0.800	0.833	0.727

### 3.4 Performance evaluation and clinical applicability analysis of the prediction model

**Figure 4:**
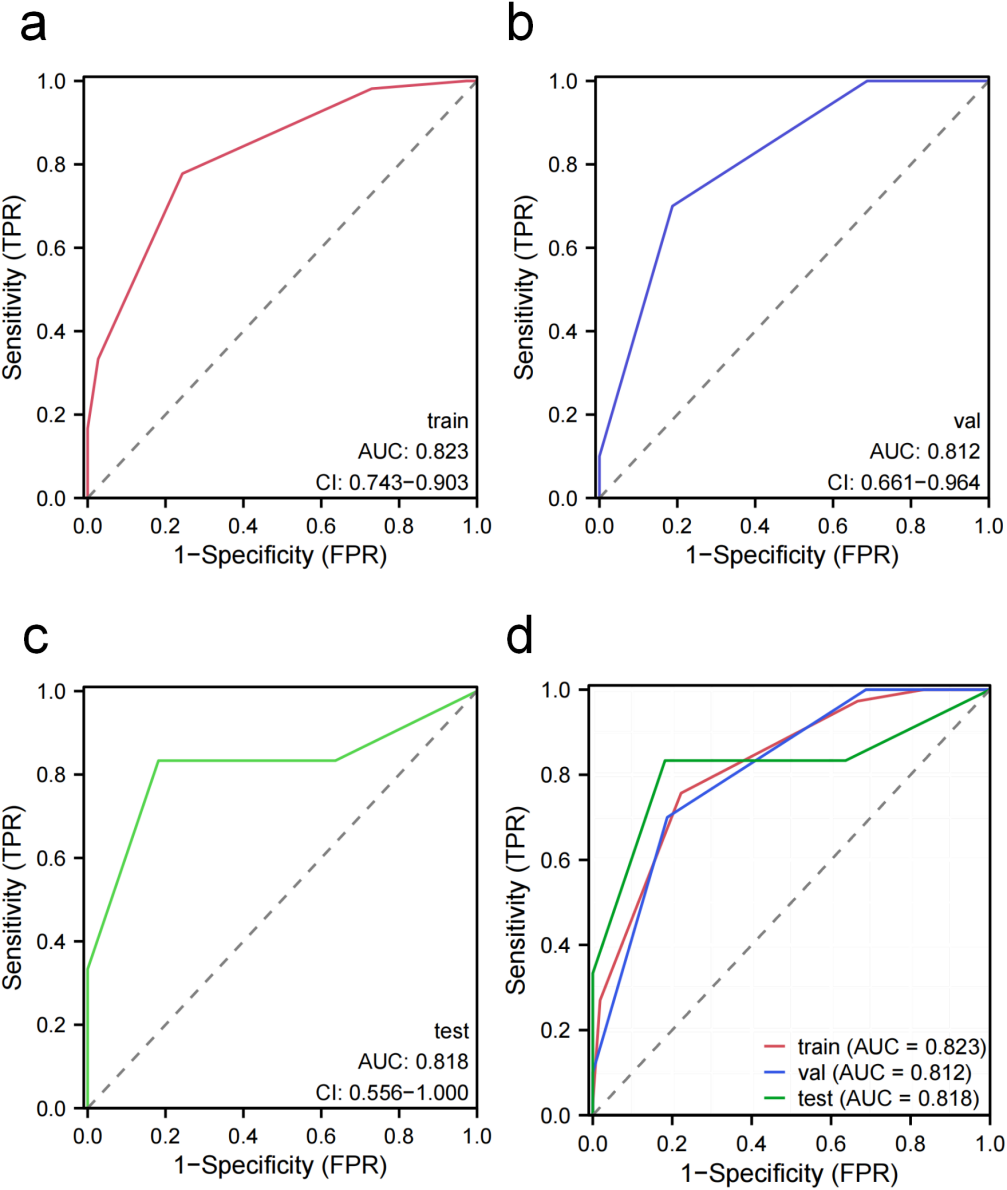
Receiver operating characteristic (ROC) curves evaluating the model's performance. **(A)** ROC curve in the training cohort, yielding an AUC of 0.823 (95% CI: 0.743–0.903). **(B)** ROC curve in the internal validation cohort, yielding an AUC of 0.812 (95% CI: 0.661–0.964). **(C)** ROC curve in the external test cohort, yielding an AUC of 0.818 (95% CI: 0.556–1.000). **(D)** Comparison of ROC curves across all three cohorts. The red, blue and green lines represent the training, internal validation, and external test cohorts, respectively. Abbreviations: AUC, area under the curve; CI, confidence interval.

ROC curve analysis showed that the radiomics-based KNN model exhibited robust discriminative performance in predicting pain relief (**Figure 4**). The model achieved a high area under the curve (AUC = 0.823, 95% CI: 0.743–0.903) in the training set. Notably, despite wider confidence intervals due to limited sample sizes, the model maintained highly consistent predictive performance in the internal validation set and external test set, with AUCs of 0.812 (95% CI: 0.661–0.964) and 0.818 (95% CI: 0.556–1.000), respectively. This cross-cohort performance stability suggests the absence of evident overfitting.

**Figure 5:**
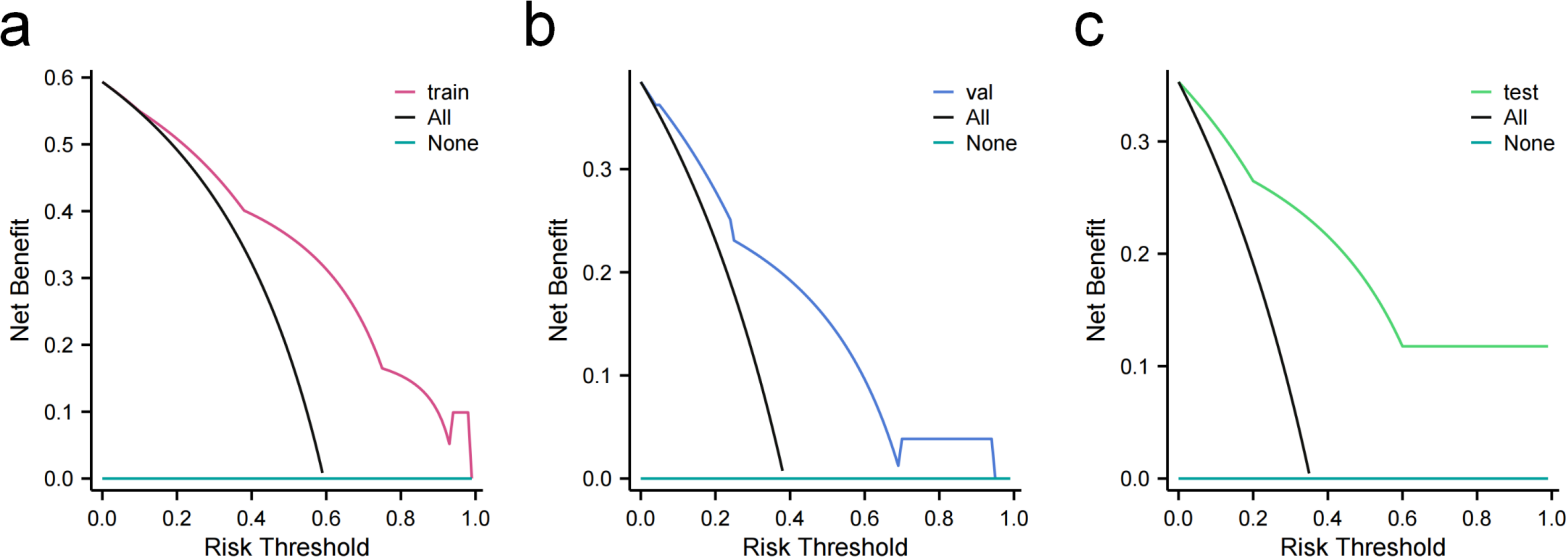
DCA of the KNN model is shown for the **(A)** training, **(B)** validation, and **(C)** test cohorts. The curves demonstrate that the KNN model yielded the highest net benefit across all three cohorts.

DCA further confirmed the clinical applicability of the model (**Figure 5**). The results demonstrated that across a broad range of threshold probabilities, the net benefit obtained from using the model consistently exceeded that of the default strategies of “treat all” or “treat none.” This indicated that the model has the potential to assist in clinically identifying patients who may benefit from pain relief through radiotherapy.

**Figure 6:**
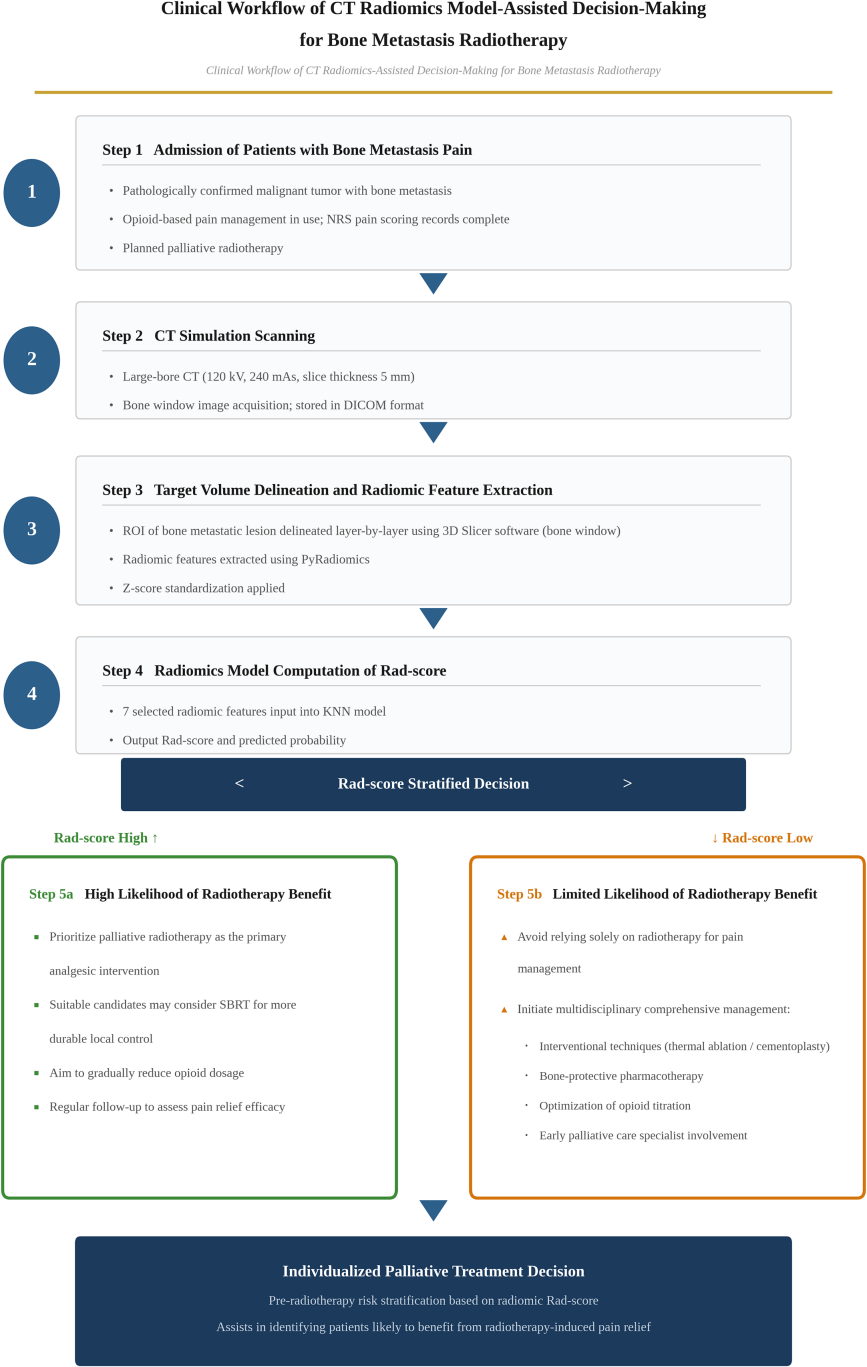
Clinical workflow of CT radiomics model-assisted decision-making for bone metastasis radiotherapy.

Based on the above performance evaluation and clinical applicability analysis, we propose a clinical workflow for applying the CT radiomics-based KNN model to assist palliative treatment decision-making in patients with bone metastasis pain (**Figure 6**).

## 4 Discussion

### 4.1 Main findings

The results of this study demonstrated that by mining radiomic features of bone metastatic lesions and integrating machine learning models, non-contrast CT images can effectively predict pain relief response following palliative radiotherapy in patients with bone metastases. Bone metastasis is one of the most common forms of distant metastasis in various advanced solid malignancies, affecting patient survival and quality of life. Radiotherapy, as a core treatment modality for pain relief in bone metastases, is widely used in palliative care and can significantly alleviate pain, promote bone stabilization, and improve functional status (2, 8, 9).Multiple studies have analyzed factors associated with pain response following radiotherapy for bone metastases, which primarily depend on the histological type of the primary tumor, performance status, prior opioid use, baseline pain scores, and radiological characteristics of bone metastases, while the choice of radiotherapy regimen has no significant impact on pain relief outcomes (6, 10, 27).

Radiomics is a convenient and efficient technique that provides a non-invasive and rapid method for assessing tumor heterogeneity and complexity by analyzing latent data within medical images (11). In recent years, radiomics techniques have shown promising potential in predicting radiotherapy outcomes. For example, the study by Cheung et al. (16) constructed a machine learning model based on CT radiomic features from 69 patients with early-stage NSCLC and pulmonary oligometastases, and found that texture features such as skewness and root mean square could effectively predict radiological response following SBRT, supporting individualized treatment planning. Similarly, in a larger-scale study, B. Lou et al. (12) enrolled 944 lung cancer patients treated with SBRT, developed a deep learning model using CT images to predict the risk of local failure following stereotactic radiotherapy, and combined clinical variables to calculate individualized radiation doses, thereby achieving image-based personalization of radiotherapy dosing.

### 4.2 Comparison with previous studies

CT radiomics has also demonstrated similar potential in other tumor types. Taking locally advanced rectal cancer as an example, Liu et al. (17) constructed and validated a radiomics model based on T2-weighted and diffusion-weighted (ADC) MRI images acquired before and after neoadjuvant chemoradiotherapy for individualized prediction of pCR in patients with locally advanced rectal cancer, achieving an AUC of 0.976 in the validation set. Subsequent studies have further confirmed and expanded upon this finding. Horvat N et al. (28) retrospectively analyzed high-resolution T2-weighted MRI images of rectal cancer patients following neoadjuvant chemoradiotherapy and indicated that the classification performance of radiomics-based quantitative features in assessing pCR (AUC 0.930) was superior to qualitative visual assessment using T2-weighted or diffusion-weighted imaging alone, as well as their combined assessment. Similar conclusions were further supported in a subsequent larger-scale multicenter study, in which Shin J et al. (29) developed and validated a radiomics model based on post-treatment T2-weighted and diffusion-weighted (ADC) MRI for predicting pathological complete response in patients with locally advanced rectal cancer following neoadjuvant chemoradiotherapy (AUC 0.820), with its classification performance significantly surpassing the visual assessment of experienced radiologists (AUC 0.740). The consistent performance of radiomics techniques in predicting radiotherapy outcomes across multiple tumor types may contribute to tumor prognosis and individualized treatment strategies, and may serve as a reliable auxiliary decision-making tool for individualized radiotherapy strategies in patients with bone metastases.

Radiomics has found potential in predicting radiotherapy outcomes across different tumor types, yet evidence for its application in the field of bone metastasis pain relief remains limited. Several recent clinical studies with methodologies similar to our study have provided important references for this issue, which are analyzed individually below. The study by Wakabayashi et al. (21) was the first to apply pre-treatment CT radiomic features to predict pain relief following radiotherapy for spinal metastases. Using a random forest algorithm to construct a radiomics model, they reported an AUC of only 0.702 for the purely clinical model, an AUC of 0.824 for the purely radiomics model, and a further improvement to 0.848 when combined with clinical features. The model performance in our study is comparable to their purely radiomics model, but with potential advantages in the following aspects: First, our study exclusively enrolled patients using opioid analgesics, which helped ensure relatively uniform pain severity among the included population, all of whom had moderate to severe pain, thereby helping to exclude confounding effects of non-opioid medications, avoiding floor effects, allowing sufficient room for NRS score reduction, and focusing on the population most in need of clinical intervention. Second, their validation approach did not reserve an independent validation sample, whereas our study retained an independent validation dataset and an external test set to verify the model’s generalizability. Third, our study compared 11 mainstream machine learning classifiers including the random forest algorithm, and random forest exhibited overfitting in our study, which suggests that algorithm selection requires caution in studies of similar scale.

However, certain discrepancies exist compared with the results of the study by Llorián-Salvador Ó et al. (20), which was based on planning CT images of 261 patients with spinal bone metastases and compared the ability of machine learning models using radiomics, semantic features, and clinical features to predict complete pain relief following palliative radiotherapy. They found that the predictive performance of the radiomics and semantic models was limited (AUC of 0.620 and 0.630, respectively), while the clinical feature model performed best (AUC 0.800). This discrepancy may be attributable to several factors: First, the endpoint definitions differed, as their study focused on complete pain relief as the primary endpoint, whereas our study used overall pain relief (CR + PR) as the endpoint, which may lead to differences in prediction difficulty and model performance. Second, their study only compared random forest and support vector machine classifiers, without conducting multi-model comparison or external validation.

The study by Fang et al. (19), which employed a CT radiomics-based machine learning model in 146 patients with tumor-induced bone metastases, was able to effectively predict pain relief outcomes following strontium-89 treatment. Bagging decision tree was used for osteolytic metastases and XGBoost for osteoblastic metastases, achieving test set AUCs of 0.889 and 0.958, respectively, which were notably higher than the AUC values in our study. This discrepancy may primarily stem from methodological factors. That study divided patients into two independent subgroups—osteolytic and osteoblastic metastases—and built separate models for each, with complex models posing a risk of overfitting on small samples, whereas our study adopted a unified modeling approach. Furthermore, that study primarily employed internal training-test splitting followed by five-fold cross-validation for internal verification, and had not yet incorporated independent external validation or multicenter data, which may further limit a comprehensive assessment of model generalizability.

### 4.3 Model selection and performance analysis

In our modeling approach, we compared 11 machine learning models (LR, Naïve Bayes, SVM, KNN, random forest, extra trees, XGBoost, LightGBM, gradient boosting, AdaBoost, MLP). Overall, the differences in model performance were notable, with tree-based ensemble models being prone to overfitting, while simpler models showed more stable generalization. The test set results best reflect true generalization capability. Logistic regression appeared optimal on the surface; however, its AUC confidence interval was relatively wide (0.864 [0.593–1.000]), which was attributable to the small test set sample size, and its perfect specificity and PPV were more likely the result of incidental correct classification of a small number of negative samples rather than robust discriminative ability. Tree-based ensemble models (such as random forest, XGBoost, and gradient boosting) achieved extremely high performance on the training set but declined on the test set, indicating a degree of overfitting. SVM and MLP showed moderately good performance but exhibited considerable fluctuation across datasets. KNN, as a typical non-parametric, instance-based learning algorithm, revealed outstanding and stable performance in this study. It maintained consistently high AUCs across the training set, validation set, and test set, with relatively balanced sensitivity and specificity, and showed no evident overfitting. Therefore, the KNN model demonstrated the best overall predictive performance in this study.

Radiomics studies commonly face the data structural challenge of high dimensionality with small sample sizes (11, 30), and the sample size of 134 cases in this study falls within a small-to-moderate scale. The study by An C et al. (31) indicated that in small-sample radiomics studies, a single random training-test split can lead to result instability, with the difference between training and test AUCs reaching as high as 0.092 ± 0.071. Relevant reviews have indicated that simple models such as logistic regression and KNN tend to be more robust than complex models under small-sample conditions. Tree-based models such as random forest, despite their excellent performance with large-sample datasets, are susceptible to overfitting when the feature dimensionality exceeds the sample size (30, 32). KNN, as an instance-based “lazy learning” algorithm, can achieve a favorable balance between bias and variance through appropriate selection of the number of neighbors (k value), without the need to fit complex decision boundaries, and has relatively lenient requirements for sample size. In this study, the KNN model achieved AUCs of 0.823, 0.812, and 0.818 in the training set, validation set, and test set, respectively. The close proximity of these three values indirectly confirms the stability of the model under the sample conditions of this study.

As a non-invasive and objective auxiliary tool, this model can assist clinicians in risk stratification of patients with bone metastases prior to radiotherapy, facilitating individualized palliative treatment decision-making. For patients with high Rad-scores, the model predicts a higher probability of pain relief following radiotherapy, and palliative radiotherapy is preferentially recommended as the core analgesic approach. For those with good performance status, SBRT may be considered to achieve more durable control, with the potential to moderately reduce opioid dosage and improve quality of life (3, 33, 34).For patients with low Rad-scores, the likelihood of pain relief following radiotherapy is limited, and radiotherapy should not be relied upon as the sole analgesic modality. Multidisciplinary comprehensive plans should be formulated in advance, incorporating interventional radiology techniques (thermal ablation, bone cement augmentation), intensified bone-protective agents, optimized opioid titration, and early pain specialist intervention (35, 36).

### 4.4 Potential biological significance of the seven selected radiomic features

(1) Higher-order filtered features based on first-order statistics.

Previous studies have provided some evidence supporting the predictive value of these first-order statistical features for radiotherapy response. In a study on radiotherapy for bone metastases from breast cancer, Kouloulias et al. directly used two first-order statistical features, mean and energy, to quantitatively assess the remineralization of osteolytic bone metastatic lesions after radiotherapy. They found that, after radiotherapy, the mean gray value of the lesions increased whereas energy decreased, suggesting that first-order image statistics are effective quantitative indicators of radiotherapy response in osteolytic metastatic lesions (37).In a study by Cheung BMF et al., 69 patients who underwent thoracic SBRT, comprising a total of 85 tumor lesions, were analyzed. The results showed that skewness and root mean square were significantly associated with post-radiotherapy imaging response, indicating that they are first-order statistical features with predictive value (16).

Of the seven radiomic features included, five were first-order statistical features. Among them, median, root mean square, maximum, and 90th percentile reflected the intensity characteristics of the lesions, whereas skewness reflected the distributional characteristics.

The feature log-sigma-1-0-firstorder-Median selected in this study represents the median value extracted from a derived image processed with a Laplacian of Gaussian filter (σ= 1.0 mm). This filter first suppresses noise through Gaussian smoothing and then performs edge detection using the Laplacian operator. A sigma value of 1.0 mm corresponds to a fine textural scale and primarily highlights local intensity variations within an approximately 1-mm radius inside the lesion. Thus, this feature reflects the central tendency of the overall signal intensity of bone metastatic lesions after fine-scale texture enhancement.

The feature log-sigma-2-0-firstorder-RootMeanSquared represents the root mean square value extracted from a derived image processed with a Laplacian of Gaussian filter (σ= 2.0 mm). This feature reflects the magnitude of local intensity variation in bone metastatic lesions after texture enhancement at an intermediate scale, corresponding to an approximately 2-mm radius. To some extent, it may be related to the overall severity of structural transition interfaces, such as the boundary between reactive osteoblastic proliferation and normal bone.

The feature log-sigma-3-0-firstorder-Maximum represents the maximum value extracted from a derived image processed with a Laplacian of Gaussian filter (σ = 3.0 mm). It reflects macrostructural characteristics within an approximately 3-mm radius in the bone metastatic lesion while suppressing finer noise and small-scale structures. The maximum density within a bone metastatic lesion may correspond to the most prominent region of density abnormality, such as an osteoblastic lesion or a focal sclerotic focus.

The feature squareroot-firstorder-90Percentile represents the 90th percentile extracted from a derived image after square root transformation. The square root transformation applies the square root to voxel intensity values. This feature reflects the high-density level of the bone metastatic lesion after compression of the dynamic range at the high-intensity end, and may help evaluate the overall high-density component of the lesion while reducing the influence of extremely high-density values.

The feature lbp-3D-m1-firstorder-Skewness represents the skewness extracted from a derived image after three-dimensional local binary pattern transformation with m = 1. It reflects the degree of asymmetry in the distribution of local textural patterns within the bone metastatic lesion. This feature may help capture asymmetry in the distribution of local texture patterns within the lesion and thereby quantify the heterogeneity of lesion microstructure in three-dimensional space, providing complementary information for the assessment of textural heterogeneity in bone metastases.

(2) Higher-order filtered features based on texture matrices.

In a study of 127 patients with stage II–III non-small cell lung cancer who underwent neoadjuvant chemoradiotherapy, Laplacian of Gaussian-filtered GLCM entropy (AUC = 0.61, p = 0.03) and root mean square were identified as independent imaging features for predicting postoperative residual tumor, whereas none of the conventional volumetric imaging features showed significant predictive value. This finding suggests that texture features may be more valuable than traditional radiologic measurements for predicting pathological response (38).In a PET-based study on the prediction of chemoradiotherapy response, three texture features—coarseness, contrast, and busyness—showed significant differences between the effective and ineffective groups, indicating the potential of NGTDM texture features for predicting chemoradiotherapy response (39).

square_glcm_Contrast represents the Contrast feature from the GLCM extracted from the derived image after square transformation. The square transformation squares the intensity value of each voxel, thereby nonlinearly amplifying the contrast between high-signal and low-signal regions in the original image. As a result, signals from high-density components are further enhanced, whereas those from low-density components are relatively compressed. Therefore, this feature reflects the spatial distribution pattern of gray-level differences between adjacent voxels in bone metastatic lesions after contrast enhancement by square transformation, and is particularly sensitive to abrupt density transitions at the interface between osteolytic and osteoblastic regions within the lesion.

wavelet_LHH_ngtdm_Coarseness represents the Coarseness feature from the NGTDM extracted from the wavelet-transformed image. This feature reflects the degree of textural coarseness in bone metastatic lesions under conditions of enhanced high-frequency detail in the y–z plane and preserved low-frequency continuity along the x direction. It may help evaluate the uniformity of transitions in tissue composition along specific spatial directions and is particularly sensitive to subtle structural variations distributed in the y–z plane.

Taken together, these findings suggest that although **Table 2** of the present study showed no statistically significant difference in the pattern of bone destruction between the pain relief and non-relief groups, five higher-order filtered features based on first-order statistics were incorporated into the radiomics score. This may indicate that radiomic features are better able than visual classification of osteolytic versus osteoblastic lesions to capture the association between imaging findings and tumor biology. The CT radiomic features of bone metastases, including gray-level distribution, textural heterogeneity, and edge-related microstructural information, may contain rich biological information about the tumor, and radiomic features extracted from computed tomography images may predict post-radiotherapy recurrence and survival outcomes (40–44).

The biological basis of this phenomenon is that quantitative features extracted via CT texture analysis reflect the status of the intratumoral microenvironment and are associated with pathological characteristics, including tumor grade, cellular hypoxia, and angiogenesis levels (45).Furthermore, image texture features demonstrate a consistent concordance with post-treatment pathological response, highlighting their potential utility in predicting treatment response. Specifically, features derived from the CT GLRLM can quantify the degree of heterogeneity in intratumoral cell density distribution and stromal composition. Meanwhile, variations in intensity features reflect the status of micro-angiogenesis to a certain extent: rapidly proliferating tumors exhibit higher image intensity due to compensatory vascular hyperplasia; conversely, when tumor growth outpaces the blood supply capacity, the resulting necrosis leads to a corresponding attenuation of intensity features. This reflects an underlying correspondence between imaging texture and histopathological evolution (46).The intensity and texture characteristics captured by these features align with tumor cell density, angiogenesis, and the extent of necrosis, thereby providing a pathological foundation for radiomics (47).

These microenvironmental features exert a direct influence on radiosensitivity (16).Tumor hypoxia represents a critical biological driver of radioresistance. In low-oxygen environments, limited oxygen availability curtails the production of reactive oxygen species (ROS), which in turn mitigates radiation-induced DNA damage and compromises radiosensitivity. CT radiomics, serving as a non-invasive tool, leverages features such as GLCM and GLRLM to characterize perfusion heterogeneity and oxygenation levels, providing a direct window into hypoxic subregions and the angiogenic landscape of the tumor microenvironment (48).Hypoxia stands as a fundamental biological driver of radioresistance, deeply intertwined with angiogenesis within the tumor microenvironment. CT-based perfusion textures have been shown to concurrently capture the heterogeneity of both processes (49). Specifically, in the context of bone metastases, recent evidence from He et al. indicates that variations in gray-scale intensity and spatial distribution may reflect the degree of osteoblastic activity and its irregular distribution within osteolytic lesions; alternatively, these radiomic signatures may manifest the diverse growth patterns and spatial extent of malignant cell infiltration (50).

In summary, these findings underscore that CT-based profiling of bone metastases serves as a non-invasive proxy for the tumor’s micro-biological landscape. Such characterization offers a compelling theoretical framework for leveraging radiomic signatures to predict radiation efficacy and guide individualized clinical decision-making.

### 4.5 Limitations

This study has certain limitations. First, although 134 patients were enrolled and an independent external test set was established, the external test set remained notably small (n = 17), representing only 12.7% of the total cohort, owing to the limited number of eligible patients treated at Center 2. This resulted in wide 95% confidence intervals for the ROC curve in the test set (0.556–1.000), and predictive stability may consequently be constrained by the limited sample size. These findings should therefore be interpreted with caution, and independent validation in a larger prospective cohort is warranted prior to clinical deployment. Second, the retrospective design of this study may introduce selection bias and information bias. Pain outcome assessment primarily relied on NRS scores documented in medical records, and NRS scores are susceptible to patients’ subjective factors, potentially leading to incomplete documentation or interpretation bias. Third, the overall response rate in the present study was 39.5%, which is lower than the 72%–75% reported for evaluable patients in previous meta-analyses that utilized non-uniform response criteria (4, 5). However, our findings are more closely aligned with the 60.4% response rate reported in a meta-analysis based on the ICPRE criteria (6). This relative consistency likely stems from our strict adherence to the ICPRE standards. Nevertheless, the response rate in our cohort remains lower than the 60.4% reported by Imano et al., a discrepancy that may be attributed to the specific clinical characteristics of the enrolled population: Although this study restricted enrollment to patients receiving opioid analgesics, with the intention of ensuring sufficient room for NRS score reduction and thereby avoiding a floor effect, the pre-treatment median NRS scores in both the relief and non-relief groups were only 2, indicating limited margin for meaningful score decline. This may have prevented some patients from meeting the criteria for PR, defined as a reduction in NRS score of ≥2 points, suggesting that radiotherapy was unlikely to demonstrate additional NRS improvement in patients whose pain had already been well controlled by opioid therapy, thereby deflating the observed relief rate and potentially compromising the sensitivity of outcome classification and the predictive performance of the model. Fourth, there were certain limitations in the selection of ROIs; for patients with multiple bone metastases, only the largest lesion was selected as a representative for feature extraction, and a prediction model constructed based solely on a single largest lesion may not adequately capture the pain heterogeneity in patients with multiple lesions. Fifth, In this study, the prediction model was ultimately constructed using radiomic features alone, without incorporating clinical variables. Although prior literature has demonstrated that combined models integrating clinical and radiomic features may yield incremental predictive value (21), clinical variables were excluded from the final model for the following reasons: (1) None of the baseline clinical characteristics demonstrated statistically significant between-group differences. This discrepancy with findings from other studies may be attributable to the strict inclusion criterion limiting enrollment to patients receiving opioid analgesics, a threshold that inherently pre-selects individuals who have already reached a level of pain severity requiring potent analgesia. This pre-selection mechanism produced a study population that was relatively homogeneous with respect to pain severity and functional status, thereby compressing between-group differences in baseline clinical characteristics and rendering them unlikely to achieve statistical significance.(2) The incremental value reported for previously published combined models has been modest and the evidence remains inconsistent, suggesting substantial heterogeneity across studies in the relative contributions of clinical variables and radiomic features, with no consistent evidence supporting the universal superiority of combined models. Specifically, Habberstad et al. (51) reported a C-statistic of only 0.69 for a purely clinical variable-based model, indicating limited inherent discriminative capacity of clinical predictors alone. Wakabayashi et al. (21) demonstrated that their combined model improved AUC by only approximately 0.024 over the radiomic-only model (0.824→0.848), representing a marginal incremental gain. In contrast, Llorián-Salvador et al. (20) found that the radiomic model exhibited limited performance (AUC 0.620) while the clinical model was superior (AUC 0.800), further underscoring this cross-study heterogeneity. Nevertheless, the exclusion of clinical variables may have constrained the predictive potential of the model to some extent. Future large-scale, multicenter studies should systematically evaluate combined modeling strategies that integrate clinical variables, radiomic features, and multimodal data, and should explore the incorporation of PET/CT metabolic information for precise identification of the responsible lesion, with the aim of further optimizing predictive performance.

## 5 Conclusion

In summary, this study constructed and validated a machine learning model to predict pain relief response following radiotherapy in patients with bone metastases. The KNN model achieved AUCs of 0.823, 0.812, and 0.818 in the training set, validation set, and test set, respectively, demonstrating the best and most stable predictive performance. The model showed potential net clinical benefit in DCA. This study provides a non-invasive imaging tool for individualized prediction of pain relief following radiotherapy in patients with bone metastases.

## Data Availability

The original contributions presented in the study are included in the article/**Supplementary Material**. Further inquiries can be directed to the corresponding author/s.
