# Supplementary material for: A CT-based radiomics model for predicting pain relief after radiotherapy in patients with bone metastases: a dual-center study
Source: Front Oncol. 2026 Apr 21;16:1813913. doi: 10.3389/fonc.2026.1813913 (PMC13138980; doi:10.3389/fonc.2026.1813913)
Supplement: Supplementary file 1 [file Table1.docx]

**Table 1. Oral MME Conversion Factors for Opioid Medications**

| Opioid Medication | Conversion Factor |
| --- | --- |
| Morphine | 1.0 |
| Codeine | 0.15 |
| Fentanyl transdermal patch (in µg/h) | 2.4 |
| Hydrocodone | 1.0 |
| Hydromorphone | 5.0 |
| Methadone | 4.7 |
| Oxycodone | 1.5 |
| Oxymorphone | 3.0 |
| Tapentadol | 0.4 |
| Tramadol | 0.2 |

**Note:** Except for fentanyl, which is measured in micrograms per hour (µg/h), all other dosages are expressed in milligrams per day (mg/day). The equivalent dose in MME can be obtained by multiplying the dosage of each opioid medication by the corresponding conversion factor.

**Examples:** Tramadol 75 mg taken 3 times daily yieclds a total daily dose of 225 mg, equivalent to 45 MME/d. Oxycodone 50 mg taken twice daily yields a total daily dose of 100 mg, equivalent to 150 MME/d. A fentanyl transdermal patch containing 4.125 mg replaced every 3 days delivers approximately 57.3 µg/h, equivalent to 137.5 MME/d. Compound preparations containing natural opium powder (e.g., Ajie tablets), whose active ingredient is morphine, where each tablet (containing 30 mg of opium powder) is equivalent to approximately 3 mg of morphine, taken 3 times daily with 2 tablets per dose, yield a total daily morphine dose of 18 mg, equivalent to 18 MME/d.

**Parenteral-to-oral conversion:** The conversion factor between oral morphine and parenteral morphine is 2.5 (i.e., 1 mg of intravenous or subcutaneous morphine = 2.5 mg of oral morphine). If any patients in the study population received intravenous or subcutaneous formulations, the dosage should first be multiplied by this factor to convert to the oral equivalent dose before being included in the baseline MEDD calculation.
