# Supplementary material for: A CT-based radiomics model for predicting pain relief after radiotherapy in patients with bone metastases: a dual-center study
Source: Front Oncol. 2026 Apr 21;16:1813913. doi: 10.3389/fonc.2026.1813913 (PMC13138980; doi:10.3389/fonc.2026.1813913)
Supplement: Supplementary file 2 [file Table2.docx]

**Supplementary Table.** Detailed Information of Radiomic Features in the Rad-score Formula

| **No.** | **Abbreviation in Formula** | **Image Filter** | **Feature Category** | **Full Feature Name** | **Coefficient** | **Description** |
| --- | --- | --- | --- | --- | --- | --- |
| 1 | *lbp_3D_m1_Skewness* | LBP-3D (m1 mapping) | First Order | Skewness | +0.0002 | Measures the asymmetry of the voxel intensity distribution. A positive value indicates a right-skewed distribution, while a negative value indicates a left-skewed distribution. Extracted after LBP-3D texture mapping. |
| 2 | *log_sigma_1_0_Median* | LoG (σ = 1.0 mm) | First Order | Median | −0.048 | The middle value of sorted voxel intensities. Extracted after Laplacian of Gaussian filtering (σ = 1.0 mm, capturing fine textures). |
| 3 | *log_sigma_2_0_RMS* | LoG (σ = 2.0 mm) | First Order | Root Mean Square (RMS) | −0.090 | Reflects the overall magnitude of voxel intensities. Extracted after Laplacian of Gaussian filtering (σ = 2.0 mm, capturing medium-scale textures). |
| 4 | *log_sigma_3_0_Maximum* | LoG (σ = 3.0 mm) | First Order | Maximum | −0.030 | The maximum voxel intensity within the ROI. Extracted after Laplacian of Gaussian filtering (σ = 3.0 mm, capturing coarse textures). |
| 5 | *square_glcm_Contrast* | Square | Texture (GLCM) | Contrast | −0.037 | Measures the intensity difference between neighboring voxels in the Gray Level Co-occurrence Matrix (GLCM). Higher values indicate greater local textural variation. Extracted after square filtering. |
| 6 | *squareroot_90Percentile* | Square Root | First Order | 90th Percentile | −0.058 | The value below which 90% of the voxel intensities fall. Reflects the distribution characteristics of high-intensity regions. Extracted after square root filtering. |
| 7 | *wavelet_LHH_ngtdm_Coarseness* | Wavelet (LHH) | Texture (NGTDM) | Coarseness | −0.070 | Measures the spatial rate of change in the Neighbourhood Grey Tone Difference Matrix (NGTDM). Higher values indicate more homogeneous textures. Extracted after wavelet LHH decomposition. |
| — | ***Intercept*** | — | — | — | +0.407 | The intercept (constant term) of the model, serving as the baseline value for the linear combination of all features. |

**Notes:**

1. Feature extraction was performed using PyRadiomics software. All feature definitions conform to the Image Biomarker Standardisation Initiative (IBSI) standards.

2. LoG = Laplacian of Gaussian; larger σ values capture coarser texture scales. LBP-3D = Local Binary Pattern 3D.

3. GLCM = Gray Level Co-occurrence Matrix; NGTDM = Neighbourhood Grey Tone Difference Matrix.

4. Wavelet LHH denotes the sub-band derived from wavelet decomposition with a low-pass filter along the x-axis, high-pass filter along the y-axis, and high-pass filter along the z-axis. Square and Square Root refer to the respective image intensity filters.

5. Coefficients were derived from a LASSO (Least Absolute Shrinkage and Selection Operator) regression model. A larger absolute coefficient value indicates a greater contribution of the corresponding feature to the Rad-score.

6. Reference: Zwanenburg A, et al. The Image Biomarker Standardization Initiative: Standardized Quantitative Radiomics for High-Throughput Image-based Phenotyping. *Radiology*. 2020;295(2):328–338.
